# Long-Term Secondary Care Costs of Endometrial Cancer: A Prospective Cohort Study Nested within the United Kingdom Collaborative Trial of Ovarian Cancer Screening (UKCTOCS)

**DOI:** 10.1371/journal.pone.0165539

**Published:** 2016-11-09

**Authors:** Mark Pennington, Aleksandra Gentry-Maharaj, Chloe Karpinskyj, Alec Miners, Julie Taylor, Ranjit Manchanda, Rema Iyer, Michelle Griffin, Andy Ryan, Ian Jacobs, Usha Menon, Rosa Legood

**Affiliations:** 1 King’s Health Economics, David Goldberg Centre, Institute of Psychiatry, Psychology & Neuroscience, King’s College London, London, United Kingdom; 2 Department of Women’s Cancer, Institute for Women’s Health, University College London, London, United Kingdom; 3 Department of Health Services Research, London School of Hygiene and Tropical Medicine, London, United Kingdom; 4 Barts Cancer Institute, Queen Mary University of London, London, United Kingdom; 5 University of New South Wales, Sydney, New South Wales, Australia; Universidade do Algarve, PORTUGAL

## Abstract

**Background:**

There is limited evidence on the costs of Endometrial Cancer (EC) by stage of disease. We estimated the long-term secondary care costs of EC according to stage at diagnosis in an English population-based cohort.

**Methods:**

Women participating in UKCTOCS and diagnosed with EC following enrolment (2001–2005) and prior to 31^st^ Dec 2009 were identified to have EC through multiple sources. Survival was calculated through data linkage to death registry. Costs estimates were derived from hospital records accessed from Hospital Episode Statistics (HES) with additional patient level covariates derived from case notes and patient questionnaires. Missing and censored data was imputed using Multiple Imputation. Regression analysis of cost and survival was undertaken.

**Results:**

491 of 641 women with EC were included. Five year total costs were strongly dependent on stage, ranging from £9,475 (diagnosis at stage IA/IB) to £26,080 (diagnosis at stage III). Stage, grade and BMI were the strongest predictors of costs. The majority of costs for stage I/II EC were incurred in the first six months after diagnosis while for stage III / IV considerable costs accrued after the first six months.

**Conclusions:**

In addition to survival advantages, there are significant cost savings if patients with EC are detected earlier.

## Introduction

Endometrial cancer (EC) is the most common gynaecological malignancy. The age standardised incidence in the UK has risen from 13.4 to 19.1 per 100,000 over the period from 1998 to 2009,[1] possibly as a consequence of rise in obesity, a known risk factor.[2] Age standardised mortality from EC has risen from 3.0 to 4.0 per 100,000 over the same period (1999 to 2012).[3] Women with EC usually present with postmenopausal bleeding and are diagnosed with early stage disease. Although five year survival is in excess of 90% in early stage, it declines sharply to 14% for those with Stage IV disease, similar to ovarian cancer patients.[4] Treatment is primarily surgical, but varies according to stage with hysterectomy and bilateral salpingo-oophorectomy (BSO) performed in women detected at Stage I, whilst for women with stage III and IV disease, chemotherapy or radiotherapy are recommended. The value of lymphadenectomy in the treatment of EC is not universally established.[5]

Understanding the costs of treating cancer, and how they vary by stage, is essential to quantify the gains from earlier detection and allow cost-effectiveness analysis of screening programmes. Costs generally increase with stage but the pattern varies across cancers. Costs of cervical cancer treatment in the UK increase rapidly from pre-invasive carcinoma (£386) through stage I (£6,623) to plateau from stages II to IV (£10,910 to £11,035).[6] Costs of breast cancer (£1991) are flat across stages I to III (£3,576, £3,996, £3,916), rising sharply to £6,590 for stage IV.[7] Data from the US indicates costs are lowest for stage IV for Colon and Rectal cancers.[8–9] Comparable data on EC is limited. A number of studies have examined the cost-effectiveness of treatment for EC.[10–14] However, these studies typically exploit short term cost data, and are often based on clinical trials with narrow inclusion criteria. Observational studies can provide data with longer follow-up that is representative of the patient population and routine practice. However, the available, observational literature on the cost of EC is based on records of patients undergoing hysterectomy (potentially excluding some late stage patients) with limited follow-up and no data on costs by stage.[15–22]

In this paper we evaluate the cost of long term management (diagnosis and treatment) and survival over five years of a population based cohort of EC patients that is broadly representative of the UK patient population and routine treatment practice. The patients were participants in the UK Collaborative Trial of Ovarian Cancer Screening (UKCTOCS), one of the largest multi-centre randomised controlled trials with follow-up through data linkage for cancer registrations and deaths.[23] The latter allowed us to identify women diagnosed with EC and calculate survival while linkage of participants resident in England to routine administrative hospital data (Hospital Episode Statistics) allowed estimation of management costs of these patients. Additional patient level covariates, derived from case note review and patient questionnaires allowed estimation of impact on costs and survival of patient and tumour characteristics.

## Methods

UKCTOCS was approved by the UK North-West Multicentre Research Ethics Committee (International Standard Randomised Controlled Trial, number ISRCTN22488978; ClinicalTrials.gov NCT00058032). Approval for the current analysis was obtained from the Joint UCL/UCLH Ethics Committee on the Ethics of Human Research (Committee A), Ref: 07/H0714/81, ethical approval granted on 11^th^ April 2013).

The main trial design, including details of recruitment and screening techniques has been detailed elsewhere.[23,24] In brief therefore, over 1.2 million women aged 50–74 in the UK were invited to be screened for ovarian cancer. Over 200,000 women were recruited between April 2001 and October 2005 through 13 centres in England, Wales and Northern Ireland. Women completed a recruitment questionnaire and provided written consent to use of their data in secondary studies. They were randomised to control (no intervention) or annual screening using either serum CA125 or transvaginal ultrasound (TVS) based strategy. All participants were followed up using their National Health Service (NHS) number through the appropriate national agencies for cancer registrations/deaths as well as by postal questionnaires. The most recent cancer registrations for this analysis were received from Health and Social Care Information Centre (HSCIC) on 17^th^ June 2014.

Women were also linked to their Hospital Episode Statistics (HES) records that were available for the period 1^st^ April 2001 to 31^st^ March 2010 for participants treated in the English NHS. HES is an electronic administrative database funded by the NHS which holds records on both inpatient and outpatient procedures for patients resident in England.[25]

### Population and data

The study population consisted of all women resident in England, enrolled in UKCTOCS, who were diagnosed with EC between April 2001 and December 2009, and for whom HES records were available. Patients with a concurrent diagnosis (synchronous) of primary peritoneal or ovarian cancer in addition to primary endometrial cancer, missing stage or incomplete HES data (possibly due to patients transferring their care outside the English NHS) were excluded.

The notification of EC diagnosis was received from multiple sources, the cancer registries (through the Health and Social Care Information Centre (HSCIC) and HES (Hospital Episode Statistics) which were searched using the ICD-10 codes of C54 (malignant neoplasm of corpus uterus) and C55 (malignant neoplasm of uterus, part unspecified), from self-reporting on the postal follow-up questionnaire or direct contact from the volunteer or their physician. Hospital records which included surgical and pathological reports were retrieved and independently reviewed by a clinician using an Outcomes Review form who confirmed EC diagnosis, stage using the FIGO classification,[26] grade and histological subtype.

UKCTOCS trial data provided patient characteristics including date of birth, Body Mass Index (BMI) collected at recruitment, hospital records of women diagnosed with EC and date of death. For confidentiality reasons only the month and year was available for the date of birth and date of death; each entry was arbitrarily assigned to the 15^th^ of the relevant month.

HES provided date of admission, date of transfer or discharge, diagnosis (as ICD-10 codes),[27] procedures undertaken (coded using the Office of Population Censuses and Surveys (OPCS) Classification of Surgical Operations version 4 codes),[28] treatment specialty, Healthcare Resource Group (HRG)[29] codes for reimbursement, and patient deprivation (measured as the Index of Multiple Deprivation (IMD) which is a geographically determined index based on areas of approximately 1500 people).[30]

We included all HES inpatient and outpatient episodes related to EC. Assignment for inpatient procedures was based primarily on OPCS-4 procedure codes taking into account the HRG code and treatment speciality. Outpatient records were less complete and procedure codes were rarely available. Outpatient records lacking procedure codes were only included where the fields coding the main specialty or the treatment speciality contained a code relating to either oncology or gynaecology. Investigations occurring within six months prior to diagnosis were included. Procedures were further classified into surgery (hysterectomy, BSO and pre-surgical investigations), adjuvant therapy, and further treatment (including management of complications).

### Estimating costs of inpatient and outpatient episodes

Costs were assigned to episodes of care on the basis of the associated HRG code.[29] This is a type of Diagnosis Related Groups (DRG) used to assign a reimbursement tariff in the English NHS. Version (3.5) was used, which allowed assignment of the 2005 Payment by Results (PbR) tariff to episodes from the entire period 2001 to 2010.[31] We then inflated the cost to 2012/13 prices using the Hospital & Community Health Services Index.[32] Finally we multiplied each cost by 1.08 which is the average Market Forces Factor for Hospitals in the English NHS.[33] Market Forces Factors are applied to payments generated from HRG codes to adjust payments for unavoidable differences in costs relating to the geographic location of each hospital.[34]

In addition to estimating cost on the basis of HRG code we adjusted costs for extended length of stay. A maximum length of stay is specified for each HRG code and length of stay beyond this point is reimbursed at a daily tariff specific to that HRG. Where patient length of stay during a spell in hospital exceeded the maximum specified for the HRG code we applied the appropriate excess bed day adjustment to the estimated cost.

PbR tariffs distinguish inpatient and outpatient procedures, and (for inpatient procedures) elective versus emergency admissions. We assumed that all inpatient admissions were elective procedures as we lacked data on the type of inpatient admission. Hospital admissions are remunerated per spell (from admission to discharge). Generally a spell consists of one episode of care, but occasionally it may consist of more than one episode. In the latter situation hospitals are remunerated according to the dominant procedure for the component episodes (typically the most expensive). We applied the costs for the most expensive episode for spells of care consisting of multiple episodes.

Inpatient episodes with missing HRG code (20%) were assigned a code on the basis of OPCS procedure codes. The majority of outpatient episodes had no HRG or OPCS code assigned. We identified records of chemotherapy and radiotherapy on the basis of the interval between admissions and the assigned treatment specialty (medical or clinical oncology). These records were assigned the appropriate outpatient PbR tariff for radiology or chemotherapy. The remaining outpatient episodes were assigned the average outpatient cost for 2012/13 of £135 per episode.[32]

### Censoring of cost data

We had missing cost data in the form of censored observations where five year follow-up date and date of death exceeded 31^st^ March 2010 (data available from HES). We had further missing data on histological subtype (0.4%), grade (3.2%), IMD (0.4%), and BMI (0.8%). IMD scores were used to partition patients into quintiles. We applied a binary classification of histological subtype which grouped Atypical Endometrial Hyperplasia (AEH) and Endometrioid EC versus all other EC histological subtypes. We used multiple Imputation (MI) to impute the missing data.[35] MI is a principled approach which fully captures the additional uncertainty generated in the imputation process.[36–37] Further details on the application of MI are provided in the supporting information (S1 File).

### Analysis of cost data

Costs arising in years following the first year after diagnosis were discounted at 3.5% according to recommendations on technology assessment by the National Institute of Health and Care Excellence (NICE).[38] FIGO stage was specified as follows: stages IA & IB; stage IC; stage II; stage III; stage IV. We subdivided costs into three categories: surgery; adjuvant therapy and further treatment as detailed above. Costs accumulated at two years after diagnosis are reported for the subset of patients with a minimum of two years follow-up in HES (diagnosis prior to 31^st^ March 2008). Total costs (after imputation of missing data) were determined for all patients at five years from diagnosis, with non-parametric 95% confidence intervals estimated from 1,000 bootstrap replicates of the data (details in appendix). Costs accumulated at five years after diagnosis for patients with complete cost data (diagnosis prior to 31^st^ March 2005) are provided in the supplementary material (Table A in S1 File).

Regression modelling was undertaken to explore the impact of patient characteristics on cost. We fitted Ordinary Least Squares (OLS) regression models to each of the three cost categories and to total costs. We pre-specified the following covariates: age, year of diagnosis, histological subtype (as Endometrioid Carcinoma/AEH or other), grade, stage, IMD quintile, Charlson score[39] and BMI. We categorised BMI as under 18.5; 18.5 to 30; over 30. Year of diagnosis was specified as the number of years following the earliest diagnosis date in the sample (January 2002). Charlson scores were determined from ICD-10 codes recorded for the hysterectomy (or the first inpatient procedure following diagnosis in the absence of hysterectomy) after exclusion of codes for cancer or metastases.

### Analysis of survival data

Dates of death were available until June 2014. Deaths were assigned to EC where the original underlying cause of death was EC (ICD-10 codes C54.0, C54.1). Regression modelling was used to investigate the impact of patient characteristics on survival over the total observation time considering both all cause and cancer specific mortality. Cox proportional hazards models were fitted to the data that adjusted for the same covariates selected for the cost analysis (detailed above). A Therneau and Grambsch test was applied to assess the appropriateness of an assumption of proportional hazards.[40]

All statistical analysis was undertaken in Stata, version 13.1.[41]

## Results

202,638 postmenopausal women aged 50–74 were randomised to UKCTOCS between April 2001 and September 2005. 157,946 of these women were resident in England. Of these women, after recruitment, notification of endometrial cancer diagnosis between 17^th^ April 2001 and 31^st^ December 2009 was received for 639 women. 148 women were excluded: 48 women on retrieving the medical notes were reviewed as having either a diagnosis of cancer other than EC or synchronous cancer of the endometrium and ovary/peritoneum; 97 with incomplete HES data (case notes indicating surgery had been undertaken but no HES records of hysterectomy or bilateral salpingo-oophorectomy (BSO); and 3 with missing data on stage. Incomplete HES data possibly arose from patients transferring their care outside the English NHS. The final cohort included 491 women of whom 479 women had undergone hysterectomy and/or BSO.

The characteristics of the women are presented in Table 1. There was a trend towards increasing age and decreasing BMI with later stage at diagnosis. However, Charlson score and deprivation show no discernible trend by stage. As expected, hysterectomy rates fell off sharply and use of adjuvant therapy increased with advancing stage. The last row in Table 1 reports five year Kaplan-Meier survival estimates by stage. There is a marked decline in 5-year survival from 94% (95% confidence interval 94.6–93.8%) for patients diagnosed with stage IA/IB to 0% (95% confidence interval 25.1% to 0%) for patients diagnosed with stage IV disease.

**Table 1:** Characteristics of patients in the study according to stage and five year survival.

	AEH^b^	Stage IA & IB	Stage IC	Stage II	Stage III	Stage IV
**No. of patients**	22	277	88	65	34	5
**Mean (SD) age (years)**	63.0 (6.2)	64.9 (6.1)	66.6 (5.8)	66.6 (5.5)	66.8 (6.2)	69.4 (3.0)
**Most deprived fifth (%)**	14%	16%	16%	15%	26%	0%
**Charlson score 1 or higher (excl. cancer) (%)**	64%	42%	31%	43%	24%	20%
**Mean (SD) BMI (kg/m**^**2**^**)**	29.6 (7.0)	29.4 (6.5)	28.9 (5.1)	28.5 (6.2)	28.3 (5.7)	27.2 (2.7)
**Proportion undergoing hysterectomy (%)**	91%	100%	99%	98%	94%	20%
**Proportion receiving adjuvant therapy (%)**	5%	9%	45%	52%	71%	80%
**Survival at five years (%)**^a^	95%	94%	89%	81%	56%	0%

^a^Kaplan-Meier estimate; SD standard deviation

^b^Atypical Endometrial Hyperplasia.

**Fig 1:**
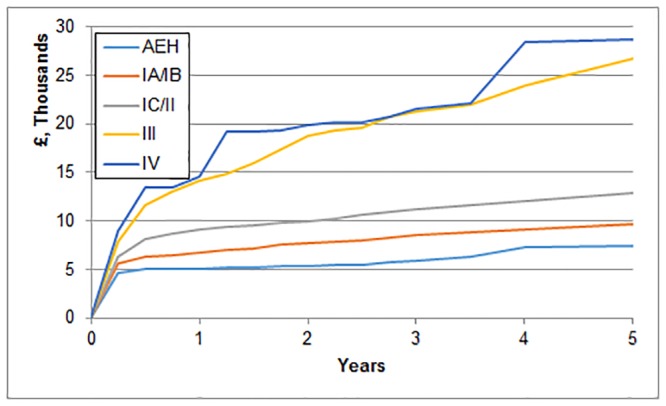
Cost accumulation over time according to stage at diagnosis.

Linking to the HES data, there were 1391 inpatient and 6501 outpatient procedures recorded for the 491 women in the cohort. Table 2 presents costs based on the HES data according to category and stage estimated at two years after diagnosis for those diagnosed prior to 31^st^ March 2008. The costs of EC treatment increased with stage. Pre-surgical investigations and surgery costs show little variation for women detected at early stage but rise for patients with stage III cancers. Costs of adjuvant therapy increase for patients when diagnosed at stage IC and rise again for patients diagnosed at stage III. Costs of further treatment also rose sharply with advancing stage. Total costs at five years post diagnosis are also reported for the entire sample after imputation of missing data (Table 2). Costs are roughly £2,000 higher than those at two years for patients diagnosed with AEH or at stage I/II. For patients diagnosed at stage III or IV costs are around £10,000 higher at five years. This pattern over time is reflected in Fig 1, which plots the mean cost accumulation over five years per patient diagnosed grouped by stage at diagnosis. For AEH, stage I and stage II cancers the majority of costs are incurred in the first six months whereas for stage III and IV cancers considerable costs are accrued after the first six months.

**Table 2:** Costs of treatment according to stage at two years post diagnosis for patients diagnosed prior to March 31^st^ 2008 and at five years for all patients after Multiple Imputation of missing data.

Stage	Costs by category at two years in 2013 £ for patients with complete cost data up to two years					Costs by category at five years in 2013 £ for all patients after imputation of missing data				
	n	Diagnosis/ Surgery	Adjuvant therapy	Further treatment	Total	n	mean	median	SD^a^	95% CI (mean)
**AEH**^b^	15	4,760	0	245	5,005	22	7,277	5,835	6,178	5,171–9,369
**IA/IB**	192	5,555	459	1,507	7,521	277	9,475	7,183	7,567	8,842–12,108
**IC**	62	5,175	3,069	1,593	9,837	88	11,707	9,117	8,095	10,147–15,546
**II**	42	5,832	2,953	2,238	11,023	65	13,965	10,185	9,931	12,154–19,793
**III**	26	7,414	5,581	5,620	18,615	34	26,080	22,342	16,296	18,417–39,062
**IV**	3	12,843	4,157	0	16,999	5	27,570	24,316	12,501	8,988–67,298
**All stages**	340	5,691	1,647	1,858	9,197	491	11,705	7,937	9,980	10,919–14,369

^a^SD standard deviation; CI confidence interval;

^b^Atypical Endometrial Hyperplasia

Linear regression of costs at two years for patients diagnosed prior to 31^st^ March 2008 indicated increased costs with increasing stage up until stage III (Table 3). Costs for stage IV were lower than those for stage III but the small number of stage IV cancers limits the generalisability of this finding. Grade showed the expected positive relationship with costs, albeit costs for grade 2 cancers were not significantly higher than costs for grade 1 cancers. There was a trend to increasing costs with increasing deprivation. Age had no impact on costs but year of diagnosis did with costs increasing by £306 (p = 0.09) a year since 2001. Linear regression of five year costs after imputation of missing data gave similar findings (Table 3). There was some evidence of lower costs in patients with a BMI>30 at two years deriving predominantly from reduced adjuvant therapy costs (-£905 for BMI>30, p = 0.013, model not reported). However, this finding was not confirmed by analysis of five year costs.

**Table 3:** Regression analysis of uncensored costs at two years and all costs at five years (after imputation) following EC diagnosis.

	Costs at two years^a^		Costs at five years^b^	
Variable	Coeff	P-value	Coeff	P-value
age (centred on 65)	3	0.94	-55	0.44
Charlson score 1	613	0.28	-108	0.90
Charlson score 2	2,482	0.24	2020	0.54
BMI<18.5	-1,141	0.66	736	0.88
BMI>30	-1,310	0.02	-1041	0.22
Stage IA/IB	1,641	0.25	812	0.69
Stage 1C	3,219	0.04	2369	0.30
Stage 2	4,348	0.007	3812	0.11
Stage 3	9,713	<0.0001	13767	<0.0001
Stage 4	8,006	0.017	12002	0.06
diagnosis year	306	0.09	1779	0.17
NS histology^c^	539	0.49	407	0.09
Grade 2	418	0.52	920	0.36
Grade 3	3,838	<0.0001	6373	<0.0001
Lowest deprivation	-1,137	0.18	-739	0.58
Low deprivation	-1,306	0.11	-840	0.51
High deprivation	712	0.40	1709	0.20
Highest deprivation	-42	0.96	-387	0.77
constant	4,848	0.003	5793	0.015

Coeff, coefficient; BMI, Body Mass Index.

^a^Costs at two years analysed for patients diagnosed prior to 31^st^ March 2008

^b^costs at five years analysed for all patients.

^c^histology other than endometrial carcinoma or Atypical Endometrial Hyperplasia

Of the 491 women included in the analysis, 61 had died at censorship with 39 having EC as primary cause of death. Follow-up time for survival ranged from 4.5 to 12.4 years with a median of 7.4 years. The Cox models for all-cause mortality and for deaths attributed to EC are presented in the supporting information (Table B in S1 File). In each case, the Therneau and Grambsch test did not reject an assumption of proportional hazards (p = 0.54). A trend to increasing mortality with increasing deprivation was observed after controlling for other patient characteristics for both all cause and cancer specific mortality. Stage, grade and histological subtype also exhibited the expected relationship. There was a significant reduction in all-cause and cancer specific mortality over the period analysed. In contrast, there was no discernible impact of BMI on mortality.

## Discussion

### Main findings

This is the first study that we are aware of that demonstrates increasing cost of treatment for EC in women diagnosed with advanced stage disease. We show that treatment costs are nearly three times as high for cancers detected at stage III compared to stage IA or IB with majority of costs for stage I / II EC incurred in the first six months after diagnosis whereas for stage III / IV considerable costs accrued after the first six months. Consequently, in addition to survival advantages there are significant cost savings if patients with EC were detected earlier. The differences in cost arise from the increased need for adjuvant therapy and increased costs of complications and follow-up procedures in patients diagnosed with advanced stage disease. These findings are important in the consideration of the cost-effectiveness of screening for EC.

### Strengths and limitations

Our study reported on EC management costs of a population based cohort of women participating in an ovarian cancer screening trial. The women are therefore more representative of those in the general population than a hospital based series. Data on treatments is drawn from a large administrative database representing clinical practice across the NHS in England, rather than any particular trial protocol. Data collected in UKCTOCS allowed access to independently reviewed stage, grade and histological subtype at diagnosis, and BMI for each woman alongside data on deprivation and comorbidities captured from HES. As a result we were able to explore the patient and tumour characteristics that have been shown to influence survival, and might be expected to influence cost.

Whilst resource use data from administrative databases is deemed to be representative of clinical practice, this source is subject to some limitations. HES only collects data on women treated by the NHS in England; women opting for privately funded treatments and those undergoing treatment in hospitals in Wales and Scotland would not have those treatments captured in HES. As a result, we observed incomplete data for 16% of women who we knew from hospital note review had surgery and had to exclude them from the analysis. Research has shown that the quality of data capture in HES is good, but it is unlikely to match that of a clinical trial [42–43]. Moreover, it is likely that not all treatments related to EC were captured and this will have resulted in underestimation of costs. It is possible that the rise in cost over the period 2002–2010 observed in the regression analysis reflects improvements in data capture in HES rather than changes in treatment protocols.

The cost estimates presented here are based on a macro-costing approach in which costs are assigned to the dominant procedure for a spell in hospital. We were able to adjust costs for excessive length of stay in hospital but we did not adjust costs for any other variation in treatments. This approach is the recommended approach for costing HES in a recent comparison of methods [44] and represents the way in which hospitals in the NHS are remunerated.

The available HES data was to the period ending 31^st^ March 2010 and consequently we did not have complete data on all women in the cohort. This is a common problem in assessing longer term costs of illness. Various methods have been developed to adjust for censoring in cost data and recover unbiased estimates of total cost [45–47]. We used a principled approach which has previously been applied to missing cost data [35,48]. Use of MI allowed regression analysis of total cost with appropriate consideration of the additional uncertainty introduced by imputation, and also allowed us to incorporate additional data on mortality (available until June 2014), an important predictor of cost.

Our analysis did not include the costs of primary care visits and referrals/all investigations as our study was limited to HES data. We did however cost all investigations documented in HES as occurring in the six months prior to diagnosis. We limited cost analysis to the five year period following diagnosis. It is possible that this underestimates costs for patients diagnosed at stages II and III who die of the disease after five years. The cost data presented here describe treatment related to EC, the impact of EC on unrelated medical costs has not been quantified [49]. We would expect this impact to be negative (cost saving) in as far as EC reduces the life expectancy of women and hence their potential to consume unrelated medical care. Finally, our analysis did not consider quality of life. Earlier diagnosis and treatment may lead to improved quality of life as well as survival.

### Interpretation

Previous studies on the cost of management of EC are predominantly from the US and have reported hospital costs for hysterectomy [15–22]. Costs range from $3790 (possibly $1996)^16^ to $36,487 (possibly $2011) [19]. Data on stage in these studies is frequently unavailable and none report costs by stage. Further limitations include short follow-up, and the use of hysterectomy to identify patients, which is likely to lead to under representation of patients with late stage disease. Our study benefitted from use of a population based cohort of EC patients, thus avoiding selection bias, detailed data on patient and tumour characteristics and long term follow-up. The cost gradient across stages is larger. The findings are important for future assessment of the cost-effectiveness of screening interventions. They indicate the potential for significant reduction in costs through earlier detection and down-staging particularly from stage III/IV to stage I/II. This is particularly relevant in view of the recent data from UKCTOCS indicating a possible reduction of ovarian cancer mortality with screening [50]. Should this reduction be confirmed on further follow up, the cost-effectiveness of ovarian cancer screening will be a driving factor in implementing a national screening programme. Although endometrial cancer screening in the general population is currently not warranted [51], earlier detection may be achieved within an ovarian cancer screening programme and therefore lead to lower costs of EC treatment.

We found a modest increase in mortality associated with lower socio-economic status, a finding in line with the broader literature on cancer survival [52,53]. In contrast to some reports from the US [54,55] we found a modest increase in resource use with increasing deprivation, which suggests that any mortality difference do not arise from access to treatment. Our study had the advantage of access to patient data on BMI and comorbidities, allowing us to control for these potential confounders. It is possible that some deaths in women with EC were wrongly attributed to cancer and our observations are attributable to elevated non-cancer mortality in deprived populations which has been previously reported in the UKCTOCS cohort [56].

## Conclusions

The cost of treating EC is strongly influenced by the stage at diagnosis and period of follow-up. Treatment costs for patients diagnosed at stage III or IV are nearly three times as high as those of patients diagnosed at stage I with a considerable proportion accruing 6 months post diagnosis. In addition to a survival benefit, there are considerable additional resource savings from diagnosing EC at an early stage, which is an important factor for assessing cost-effectiveness of EC screening.

## Supporting Information

S1 File Table A in S1 File. Costs of treatment according to stage at five years for patients with complete cost data (diagnosis prior to March 31^st^ 2005). Table B in S1 File. Cox analysis of all cause and cancer specific mortality (after imputation).
(DOCX)
