# Supplementary material for: Long-Term Secondary Care Costs of Endometrial Cancer: A Prospective Cohort Study Nested within the United Kingdom Collaborative Trial of Ovarian Cancer Screening (UKCTOCS)
Source: PLoS One. 2016 Nov 9;11(11):e0165539. doi: 10.1371/journal.pone.0165539 (PMC5102347; doi:10.1371/journal.pone.0165539)
Supplement: S1 File — Table A in S1 File. Costs of treatment according to stage at five years for patients with complete cost data (diagnosis prior to March 31st 2005). Table B in S1 File. Cox analysis of all cause and cancer specific mortality (after imputation). (DOCX) [file pone.0165539.s001.docx]

# Supporting information

Administrative censoring of longitudinal cost data is commonly encountered and the potential bias it generates has been documented.^47^ We had access to HES data up to 31^st^ March 2010, resulting in a considerable number of patients with incomplete follow-up. Methods to adjust for administrative censoring include Lin’s product-limit estimator^45,46^ and MI.^35,48^ MI is a principled approach which fully captures the additional uncertainty generated in the imputation process.^36,37^ MI replaces missing data with a set of *M* plausible values, each of which are generated from the conditional distribution of the missing observations given the observed data. Each of the resulting *M* datasets is analysed and statistics are combined using Rubin’s rules.^36^ MI is valid under an assumption that the data is Missing at Random (MAR) or ‘ignorable’ which means that missingness is dependent on covariates but not on the outcome variable (cost). We undertook MI using chained equations with 50 imputations. We imputed total quarterly costs for each quarter of the first three years where patients had missing data on resource use. For year four we imputed half yearly costs, and yearly costs in year five for any patients with incomplete follow-up. We imputed grade and IMD quintile using ordered logistic regression. Histological subtype was imputed using logistic regression. Missing cost data and BMI were imputed using Predictive Mean Matching to allow for non-normal distributions of the data.^58^

In order to generate non-parametric confidence intervals the raw data was bootstrapped and the MI procedure undertaken on each of 1,000 bootstrap replicates. Mean costs, by stage at diagnosis, were calculated for each of the 50 imputations of the 1,000 replicates. Non-parametric 95% confidence intervals were specified as 1251^st^ and 48750^th^ values of the distribution of all 50,000 means.

|  |  | Costs (2013 £) | | | |
| --- | --- | --- | --- | --- | --- |
| Stage | n | Diagnosis/  Surgery | Adjuvant therapy | Further treatment | Total |
| AEH | 7 | 5,035 | 2,647 | 1,767 | 9,449 |
| IA/IB | 68 | 5,583 | 417 | 2,190 | 8,190 |
| IC | 16 | 5,548 | 3,582 | 2,034 | 11,164 |
| II | 14 | 5,795 | 2,624 | 3,192 | 11,611 |
| III | 12 | 8,571 | 5,588 | 9,856 | 24,016 |
| IV | 1 | 21,314 | 967 | 0 | 22,281 |
| All stages | 118 | 6,008 | 1,771 | 3,024 | 10,803 |

Table A. Costs of treatment according to stage at five years for patients with complete cost data (diagnosis prior to March 31^st^ 2005).

|  | All-cause mortality |  | Deaths attributed to cancer |  |
| --- | --- | --- | --- | --- |
| Variable | hazard ratio | p value | hazard ratio | p value |
| age | 1.004 | 0.85 | 1.024 | 0.49 |
| age squared | 0.995 | 0.09 | 0.990 | 0.04 |
| Charlson score 1 | 1.048 | 0.86 | 0.921 | 0.83 |
| Charlson score 2 | 2.276 | 0.31 | 7.603 | 0.02 |
| BMI<18.5 | 2.849 | 0.19 | * | * |
| BMI>30 | 1.001 | 1.00 | 0.797 | 0.56 |
| Stage IA/IB | 0.989 | 0.99 | * | * |
| Stage 1C | 1.610 | 0.67 | 0.623 | 0.44 |
| Stage 2 | 1.763 | 0.61 | 1.376 | 0.51 |
| Stage 3 | 5.020 | 0.15 | 4.941 | 0.00 |
| Stage 4 | 19.631 | 0.02 | 7.303 | 0.02 |
| diagnosis year | 1.022 | 0.74 | 1.063 | 0.52 |
| NS histology | 2.339 | 0.002 | 2.805 | 0.01 |
| Grade 2 | 1.426 | 0.39 | 6.849 | 0.07 |
| Grade 3 | 4.898 | <0.0001 | 29.412 | <0.0001 |
| Lowest deprivation | 0.803 | 0.59 | 0.906 | 0.87 |
| Low deprivation | 0.915 | 0.81 | 0.901 | 0.85 |
| High deprivation | 1.452 | 0.30 | 1.448 | 0.47 |
| Highest deprivation | 1.111 | 0.79 | 1.046 | 0.94 |

*insufficient deaths to estimate coefficient; dummy excluded

Table B. Cox analysis of all cause and cancer specific mortality (after imputation)
